# Maize, Peanut, and Millet Rotations Improve Crop Yields by Altering the Microbial Community and Chemistry of Sandy Saline–Alkaline Soils

**DOI:** 10.3390/plants13152170

**Published:** 2024-08-05

**Authors:** Liqiang Zhang, Jianguo Zhu, Yueming Zhang, Kexin Xia, Yuhan Yang, Hongyu Wang, Qiuzhu Li, Jinhu Cui

**Affiliations:** College of Plant Science, Jilin University, Changchun 130012, Chinajgzhu21@mails.jlu.edu.cn (J.Z.); kxxia21@mails.jlu.edu.cn (K.X.); wakk47640887@163.com (Y.Y.); hong_yu@jlu.edu.cn (H.W.)

**Keywords:** sandy saline-alkaline soils, crop rotation, soil microorganisms, yield economic benefits

## Abstract

Crop rotation increases crop yield, improves soil health, and reduces plant disease. However, few studies were conducted on the use of intensive cropping patterns to improve the microenvironment of saline soils. The present study thoroughly evaluated the impact of a three-year maize–peanut–millet crop rotation pattern on the crop yield. The rhizosphere soil of the crop was collected at maturity to assess the effects of crop rotation on the composition and function of microbial communities in different tillage layers (0–20 cm and 20–40 cm) of sandy saline–alkaline soils. After three years of crop rotation, the maize yield and economic benefits rose by an average of 32.07% and 22.25%, respectively, while output/input grew by 10.26%. The pH of the 0–40 cm tillage layer of saline–alkaline soils decreased by 2.36%, organic matter rose by 13.44%–15.84%, and soil-available nutrients of the 0–20 cm tillage layer increased by 11.94%–69.14%. As compared to continuous cropping, crop rotation boosted soil nitrogen and phosphorus metabolism capacity by 8.61%–88.65%. Enrichment of Actinobacteria and Basidiomycota increased crop yield. Crop rotation increases microbial community richness while decreasing diversity. The increase in abundance can diminish competitive relationships between species, boost synergistic capabilities, alter bacterial and fungal community structure, and enhance microbial community function, all of which elevate crop yields. The obtained insights can contribute to achieving optimal management of intensive cultivation patterns and green sustainable development.

## 1. Introduction

Soil salinization is a growing global problem, with about 11 million ha of saline land [1]. The Songnen Plain in Northeast China is one of the three largest saline soil areas in the world. The existing saline land area in Jilin Province is 3.733 million ha, of which the moderate and severe salinized land accounts for 67.4% of the total saline land area. The area of severe salinized soil is still expanding at a rate of 1.4% per year [2]. Although sealing, grazing, and application of chemical amendments can improve medium and light saline soils, these methods have problems, such as slow improvement speed and low economic benefits for dryland saline soils [3]. In the context of the global food crisis, saline land played an important role as a reserve in ensuring national food security [4].

Intensive cultivation pattern is one of the most direct and critical factors affecting the physical, chemical, and biological properties of soils. Crop rotation is an important means to alleviate the “continuous cropping obstacle”, rationalize the use of soil nutrients, and coordinate the uptake of nutrients by crops [5]. Therefore, understanding the impact of crop rotation on maize is essential for developing nutrient management strategies to optimize yields while maintaining the sustainability of cropping patterns. In recent years, affected by millet prices and intensive cultivation, farmers in the main distribution area of saline land in Jilin Province mostly chose to plant maize as the main crop, owing to better economic benefits and a higher degree of machinery use [6]. However, soil salinization is becoming increasingly severe due to year-round continuous cropping and excessive chemical fertilizers application, hindering the improvement of crop yield and quality [7]. The rotation of leguminous and gramineous crops is the essence of traditional agriculture. It can effectively solve the problems arising from continuous cropping, maintain soil health, increase soil microbial abundance, improve the efficiency of nitrogen and other nutrients usage, reduce the application rates of chemical fertilizers, and promote high yield [8]. However, the effect of crop rotation measures on saline soil properties and crop yields, especially the contribution of soil microorganisms, is not well investigated.

A good soil environment is the foundation for high and stable crop yield and is a biological metric that combines land use and land nourishment [9]. Previous studies proved that crop rotation can increase the effectiveness of saline soil nutrients and improve the structure of rhizosphere microbial communities [10,11]. Ma et al. (2023) reported that crop rotation is favourable to soil aggregate stability as compared to single continuous cropping [12]. The degree of influence of crop rotation on soil aggregate stability becomes more obvious with the prolongation of crop rotation years, as evidenced by a decrease in the soil compactness, the increase in clay millet content, and the enhancement of the degree of discrete particles. With the increase in soil depth, the soil bulk weight and field water holding capacity increased, but the capillary porosity and soil-saturated hydraulic conductivity exhibited a stable decreasing trend [13].

Crop rotation can increase soil fertility, natural processes occurring in the soil, such as nitrogen fixation by leguminous plants, and influence the growth and development of the following crop by elevating the nitrogen content of the soil and improving the soil structure by adding soil organic matter [14]. Many studies found that under crop rotation conditions, soil pH and available nutrient content in the 0–20 cm soil layer decreased to varying degrees, soil nutrient and organic matter depletion increased, and the effectiveness of soil nitrogen and phosphorus nutrients and their activation rates increased significantly [15,16,17].

Soil microorganisms can be an important indicator of changes in soil quality in saline soils [18]. Crop rotation can cause changes in microbial communities, and different species exuding different types of root secretions can sculpt the structure of soil bacterial communities [19]. It was proved in common farmland that the implementation of appropriate crop rotation can effectively improve the quality of the soil environment, enhance soil microbial activity, and increase the diversity of soil microbial communities and populations [20]. However, it is not clear whether the same rules apply to salinized farmland ecosystems caused by crop rotation.

Monoculture planting of salt-tolerant plants is currently the major technique to ameliorate saline–alkaline land in China. The traditional monoculture agricultural production system of planting oil sunflower and maize is used in dry areas [21]. However, long-term monoculture farming methods can cause problems such as shallow soil plowing layer, soil organic matter enrichment in the surface layer, high soil bulk density, and frequent occurrence of pests and diseases, which pose a serious threat to the normal growth and development of crops, and adversely affect crop yields [22]. Based on the above, it can be presumed that crop rotation after a rational combination of different kinds of crops can fully utilize the nutrients at different depths of saline soils, and thus, the crop rotation pattern best suited for local conditions can be determined. Maintaining the nutrient balance of saline soils, the coordination of water and fertilizers, sustainable land use, and the ecological reconstruction of saline soils are all critical.

Building upon the aforementioned literature, it was postulated that the alterations in cropping patterns caused by crop yield in maize, peanut and millet rotations might be closely linked to key species within the soil’s chemistry properties and microbial community. To confirm the hypothesis, saline soil (0–40 cm) was sampled at maturity in a maize–peanut–millet rotation for three consecutive years. The soil chemical attributes were quantified and high-throughput sequencing was used to characterize the composition of bacterial and fungal communities. The objectives of this study were to (1) investigate the effects of crop rotation on soil chemistry properties in the 0–20 cm and 20–40 cm soil layers during crop maturity; (2) elucidate the main influencing factors and mechanisms of microbial changes in saline soils following crop rotation; (3) predict the theoretical causes of the changes in metabolic functions from the microscopic point of view; and (4) ascertain whether crop rotation could be used as a saline soil amelioration measure to improve economic efficiency and clarify the interaction between soil microenvironment and crop yield.

## 2. Materials and Methods

### 2.1. Field Site

A long-term positioning field experiment for a maize–peanut–millet rotation system was set up in May 2020 in Haladao Village, Tongyu County, Jilin Province (126.35° N, 44.50° E). At an elevation of 156.4 m above sea level, the area receives an average annual precipitation of 450 mm (80% of the rainfall occurs in summer), and an annual evapotranspiration of 1600 mm. The saline soil in the area has a water content of about 18%, and the average annual temperature ranges from 1 to 14 °C, with a maximum of 38 °C and a minimum of −28 °C. It harbors typical sandy saline–alkaline soils, and the local weather conditions produce seasonal frost fluctuations on its saline–alkaline soil structure. Before the experimental initiation, the soil metrics were: pH8.49, organic matter 1.17%, total nitrogen 580.6 mg kg^−1^, available phosphorus 3.75 mg kg^−1^, and available potassium 50.55 mg kg^−1^.

### 2.2. Experimental Design

**Figure 1:**
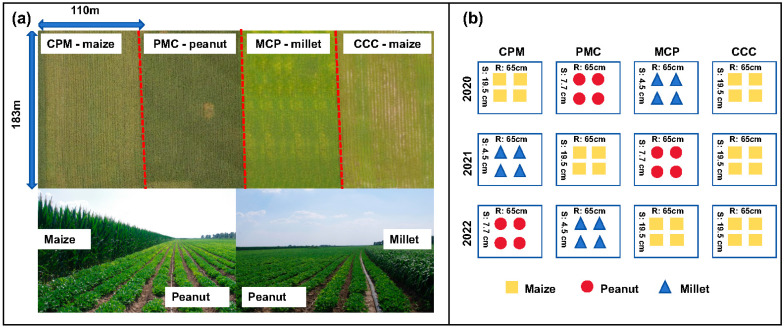
Layout of the experiment’s cropping modes and arrangement of plants. Maize–peanut–millet rotations (CPM), peanut–millet–maize rotations (PMC), millet–maize–peanut rotations (MCP), and maize continuous cropping (CCC). (**a**) is an aerial view and two photographs of the experimental site, (**b**) enlists the crops planted in each treatment for both rotation and continuous cropping in 2020–2022, and the numbers between the two columns are the spacing of the rows (R), and the numbers between the two rows are the spacing of the plants (S).

The experiment was set up as a large-scale production trial with two cropping strategies, namely maize–peanut–millet triple rotation and maize continuous cropping, with continuous cropping (CCC) serving as the control treatment in 2019. The previous crop of the test plot was maize, with the maize variety ‘Fumin 985’, the peanut variety ‘Baiyuanhua 9’, and the millet variety ‘Jiugu 32’. The experiment was conducted in four equally sized experimental plots. Each experimental plot was 2 ha, three of which were planted in a continuous maize–peanut–millet rotation and the remaining one in a continuous maize. The row spacing was 65 cm and the maize sowing density was 78,000–79,000 plants ha^−1^, with a seedling retention of plants 70,000 plants ha^−1^. The peanut sowing density was 180–200,000 plants ha^−1^, and the millet sowing density was 350,000–360,000 plants ha^−1^. The soil fertilizer was applied with 560 kg ha^−1^ of nitrogen–phosphorus–potassium compound fertilizer (N:P:K = 26:15:15) and 400 kg ha^−1^ of urea (N ≥ 46%) in two subsequent applications (Figure 1); for all the abbreviations used and their explanations, see Abbreviations part for details.

### 2.3. Sampling

For analysis, soil samples were collected on 29 September 2019, 30 September 2020, 29 September 2021, and 26 September 2022 (the above are maturity stage). Five points (“S” distribution) were selected in each experimental area, and the collection equipment was sterilized beforehand. Soil was sampled at two plough depths, 0–20 cm and 20–40 cm, respectively. Fresh soil samples were quickly placed in ice boxes after removing impurities such as gravel and plant residues. Some were air-dried for chemical analyses, while others were stored in a refrigerator at −80 ℃ for microbial diversity measurements. The 2019 soil samples (CK) were only assessed for soil chemical properties.

### 2.4. Measurements and Methods

#### 2.4.1. Crop Yields

When the crop (maize, peanut, and millet) ears were ripe, 100 m^2^ were collected from each plot. The kernels were threshed followed by weighing the grain, and yield was calculated per hectare of the area harvested. Three replicates were used to determine the moisture content of kernels using a moisture meter (50 MHz, Kett, Tokyo, Japan), and yield was reflected as the grain’s weight at a moisture content of 14%.

#### 2.4.2. Physico-Chemical Attributes of Soil

For extraction, the 1:5 soil–water ratio samples were stirred for 5 min at 180 rpm and left for 0.5 h, followed by pH [23] and EC measurements. Available soil nitrogen (AN) and available soil phosphorus (AP) fractions were extracted with 10 mM CaCl_2_ (1:5 soil–solution ratio) and 0.5 M NaHCO_3_ (1:20 soil–solution ratio), followed by stirring at 180 rpm for 2 h. After being still for 0.5 h, these were passed through PES filter membranes (0.45 µm) [24]. The Kjeldahl method and acid digestion methods were used to determine total soil nitrogen (TN), potassium (TK), and total phosphorus (TP), respectively. The PES-filtered aliquots were evaluated by a flow analyzer (AutoAnalyzer 3, CN) [25]. The soil-available potassium (AK) fraction was extracted with ammonium acetate, stirred for 2 h at 180 rpm, and left still for 0.5 h. The PES-filtered samples were subjected to flame photometry (FP6400, Shanghai, China). The volumetric method of potassium dichromate dilution calorimetry was used to quantify the soil organic matter (SOM) fractions [26].

#### 2.4.3. High-Throughput Sequencing

A DNA kit (MN NudeoSpin 96 Soi) was used for total DNA extraction and yield was quantified via NanoDrop. The primer pair for bacterial 16S comprised 338F (5′-ACTCCTACGGGAGGCAGCAG-3′) and 806R (5′-GGACTACHVGGGTWTCTAAT-3′), while ITS1F (5′-CTTGGTCATTTAGAGGAAGTAA-3′) and ITS2 (5′-GCTGCGTTTCTTTCATCGATGC-3′) were used for fungal 18S rRNA. The PCR cycles included 5 min pre-denaturation at 94 °C, 30 cycle amplification at 94 °C, 50 °C each for 30 s, and 72 °C for 60 s, followed by a 7 min extension run at 72 °C. The obtained aliquots were stored at 4 °C and agarose gel electrophoresis was run thereafter to visualize DNA.

Library construction went as follows: (1) ligation of the ‘Y’ junction; (2) bead screening to eliminate self-associated fragments; (3) library enrichment via PCR; and (4) single-stranded fragments generation after alkali denaturation.

Next, these eight sequencing steps were applied: (1) fixing on the chip of the DNA fragment complementary to the primer; (2) a bridge formation with the other end, randomly complementary to another primer; (3) PCR; (4) single strand generation via linearization; (5) syntheses of 1 base per cycle via labelled dNTP and DNA polymerase; (6) laser scanning to read the nucleotide sequence generated in the first round; (7) repeating polymerization cycles; and end by (8) recorded fluorescence signals interpretation to decipher the DNA fragment sequence [27].

### 2.5. Statistical Assessments

Statistical significance was evaluated using SPSS 22.0 software. The effects of crop rotation on soil parameters were analyzed by one-way ANOVA. The Duncan test and a post-hoc test further validated the critically different means. To negate false positives, the false discovery rate (FDR) was tracked carefully. Principal component analysis (PCA) was used to examine the critical soil factors that influenced the changes in the chemical properties of saline soils. To assess the relationships between the relative abundance of dominant microorganisms, soil chemical properties, and yield, Pearson’s correlation was used. The Bray–Curtis coefficient examined the beta diversity. To assess the functions of soil bacteria and fungi subjected to varied treatments and screen the expression level of nutrient metabolism-related enzyme genes, phylogenetic investigation of communities by reconstruction of unobserved states (PICRUSt) was deployed.

Visual analysis of microbial ecological networks and derivation of topological indices were made using Gephi software (v0.9.6). The following topological indices were used to describe the nodes and connecting lines in the constructed microbial network: (1) the number of connecting lines of a node, which is the sum of all lines linked to each node; (2) the median centrality of a node, which is the node located on the shortest path between two nodes, this calculated according to the formula in (a) below; (3) the topological coefficient of a node, which conveys the proximity of nodes and is expressed by formula (b) below; (4) the connecting line weight, which reflects the number of connections between a particular operation taxonomic unit (OTU) node and the sample node; and (5) the connecting line centrality, a parameter that gauges the importance of each connecting line in the information transfer process of the whole network [28].

Structural equation modeling (SEM) of the direct or indirect effects of crop rotation on yield pathways was implemented using R v4.3.1 (https://www.r-project.org/, 16 May 2024).


(1) $$Cb\left(n\right)=\sum_{s\neq n\neq t}\left(\frac{\sigma_{st}\left(n\right)}{\sigma_{st}}\right)$$

In the above equation, *n* is the destination node; *s* and *t* are nodes in the network other than *n*; $\sigma_{st}$ denotes the number of shortest paths from node *s* to node *t*; and the term $\sigma_{st}(n)$ denotes the number of shortest paths from node *s* to node *t* that must pass through node *n*.


(2) $$T_{n}=\frac{avg\left(J_{\left(n,m\right)}\right)}{k_{n}}$$

Here, $J_{\left(n,m\right)}$ is the number of all nodes adjacent to both nodes *n* and *m*, where the value of $J_{\left(n,m\right)}$ is increased by 1 if *n* is directly adjacent to *m*; and $k_{n}$ is the number of all neighbours of that node.

## 3. Results

### 3.1. Statistical Analysis

**Figure 2:**
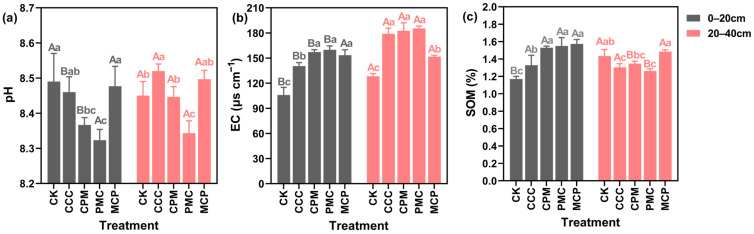
Effects of different cropping systems on soil pH (**a**), EC (**b**), and organic matter (SOM) (**c**). CK is for the 2019 continuous cropping treatment. CCC is for 2020–2022 continuous cropping treatment, CPM, PMC, and MCP are for 2020–2022 rotation treatment, where C is for maize, P is peanut, and M is for millet. The bars represent the mean ± SE, n = 45 (CK, n = 15) replicates. Capital letters (A, B) on the error line denote differences between soil depth (0–20 cm and 20–40 cm) under the same treatment (*p* < 0.05). Small letters (a–c) on the error line indicate differences between treatments under the same soil depth (*p* < 0.05).

At 0–20 cm depth, soil EC and organic matter (SOM) content of each treatment were higher than that of CK treatment, increasing by 30.09–48.12% and 13.41–34.12%, respectively, as illustrated in Figure 2. Soil pH of PMC (peanut) was reduced by 2.36% compared with that of CK treatment (*p* < 0.05). Except for soil pH, both EC and SOM contents were raised in the rotational crop treatments compared to the continuous cropping treatments (CCC), with increases of 8.43–12.17% and 13.44–15.84%, respectively, and pH was slightly decreased, but the decline was not significant. At 20–40 cm depth, the treatments increased soil EC by 15.44–30.80% compared to the CK treatment. The PMC treatment had the lowest pH and SOM content in this soil layer, which was lower than the CK treatment by 0.84% and 16.03%, respectively. The MCP (millet) treatment reduced EC and SOM by 17.97% and raised SOM by 12.18% as compared to the CCC treatment (*p* < 0.05).

In each crop rotation treatment, the variations in pH, EC, and SOM in each soil layer exhibited a specific pattern with the change in crops planted. For example, in the 0–20 cm and 20–40 cm soil layers, the lowest pH and SOM values, and the highest EC values were observed in the PMC crop, whereas the opposite was recorded in the MCP crop. When CCC and CK treatments were compared, pH declined and EC and SOM increased at 0–20 cm depth, whereas SOM content decreased and pH and EC elevated at 20–40 cm depth with increasing years of CCC. Overall, the three-year crop rotation (2020–2022) increased SOM in the 0–20 cm soil layer and decreased pH in the 0–40 cm depth. Although SOM was raised in the CCC treatment in the same year, pH and EC also increased, and soil salinity deteriorated.

### 3.2. Soil Nitrogen, Phosphorus and Potassium

**Figure 3:**
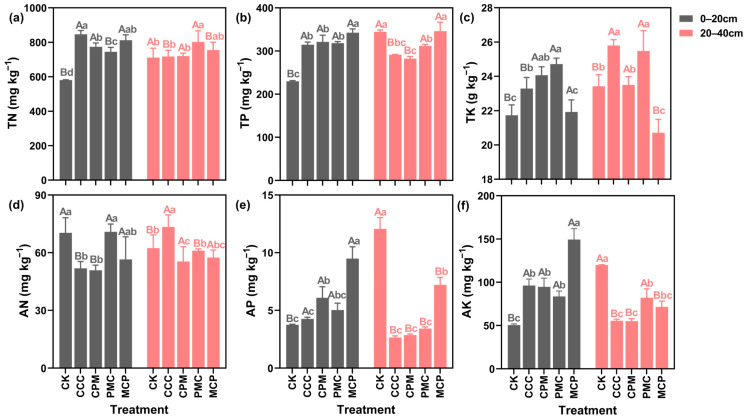
Effects of different cropping systems on soil total nitrogen (TN) (**a**), total phosphorus (TP) (**b**), total potassium (TK) (**c**), available nitrogen (AN) (**d**), rapidly available phosphorus (AP) (**e**), and available potassium (AK) (**f**). CK is for the 2019 continuous cropping treatment. CCC is for 2020–2022 continuous cropping treatment, CPM, PMC, and MCP are for 2020–2022 rotation treatment, where C is for maize, P is peanut and M is for millet. The bars represent the mean ± SE, n = 45 (CK, n = 15) replicates. Capital letters (A, B) on the error line indicate differences between soil depth (0–20 cm and 20–40 cm) under the same treatment (*p* < 0.05). Small letters (a–c) on the error line indicate differences between treatments under the same soil depth (*p* < 0.05).

In the soil at 0–20 cm depth, the TN, TP, and TK contents of all the treatments were higher than that of the CK treatment (Figure 3a–c). When compared to the CCC treatment, each of the rotation treatments decreased the soil TN content, with the highest reduction of 12.02% in the PMC treatment. The TP content was higher in rotation than in continuous cropping. In the MCP treatment, it was 8.10% higher than the CK treatment. However, the TK content was 5.85% lower than that in the CCC treatment. In the context of 20–40 cm depth, the TP content of the soil in each treatment was lower than that in the CK treatment, and the most reduction of 18.01% was observed in the CPM treatment. Except for the MCP treatment, the TK content of the other treatments was higher than that of the CK. Further, though TN content slightly increased, it was not significantly different in all treatments (*p* > 0.05).

As seen in Figure 3d–f, the AN in the soil was lower in all treatments at 0–20 cm depth, with a reduction of 38.15% and 34.02% in CCC and CPM treatments, as compared to CK treatment. Nevertheless, the AP and AK contents of the treatments were higher than those of the CK treatment by 11.94–60.46% and 39.45–69.14%, respectively. At 20–40 cm depth, the AN content in CCC was higher than that in CK treatment, while its content in CPM, PMC, and MCP was lower than that in CK. When compared to CK treatment, the AP and AK contents of each treatment were lower by 43.35–79.17% and 31.57–54.09%, respectively. The planted crop has a strong influence on soil nutrient changes. For example, the CCC and CPM treatments, both planted with maize, exhibited similar changes in soil nutrient content at different depths. However, the MCP treatment showed higher soil phosphorus content than the other treatments.

### 3.3. Principal Component Analysis of Soil Chemical Properties

To clarify the main factors influencing the productivity of wind–sand–saline soils, each soil chemical attribute was subjected to PCA. As shown in Table 1, five factors, i.e., soil EC, pH, AK, SOM, and AN, explained 96.08% of the variation in soil chemical properties, in the order of EC > pH > AK > SOM > AN. These five factors were the most critical soil chemistry parameters that influenced changes in the productivity of saline soils. Therefore, EC and pH in soil are important factors affecting the productivity of wind–sand–saline soils.

### 3.4. Changes in Soil Microbial Communities

#### 3.4.1. Alpha Diversity of Bacterial and Fungal Communities

**Figure 4:**
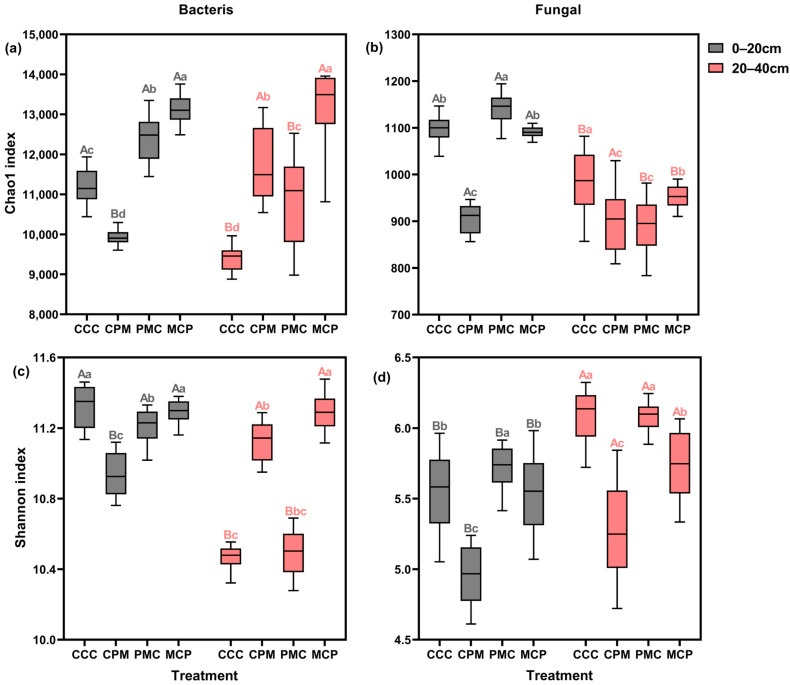
Effects of different cropping systems on alpha diversity of bacterial (**a**,**c**) and fungal (**b**,**d**) communities. Chao1 index (**a**,**b**) and Shannon index (**c**,**d**). CCC is for 2020–2022 continuous cropping treatment, CPM, PMC, and MCP are for 2020–2022 rotation treatment, where C is for maize, P is peanut, and M is for millet. The box plot has the upper and lower quartiles at each end, the horizontal line in the middle indicates the median, and the lines connecting the two ends are the minimum and maximum values (n = 15). Capital letters (A, B) on the boxes’ maximum values line indicate differences between soil depth (0–20 cm and 20–40 cm) under the same treatment (*p* < 0.05). Small letters (a–c) on the error line indicate differences between treatments under the same soil depth (*p* < 0.05).

To evaluate the alpha diversity of individual soil samples, the abundance (chao1) and diversity (Shannon) of soil bacteria and fungi were calculated for each treatment (Figure 4a–d). Both CPM and MCP treatments exhibited higher bacterial and fungal abundances than the CC treatment at 0–20 cm depth, with an average increase of 14.02% and 7.74%, respectively. Both bacterial and fungal abundances were lower in the PMC treatment than in the CC treatment, with reductions of 9.51% and 17.15%, respectively. Amidst all treatments, bacterial and fungal community diversity was lowest in the PMC treatment, with the overall bacterial diversity trend of CCC > MCP > CPM > PMC. There was no significant difference in fungal community diversity between treatments.

Bacterial abundance and diversity varied similarly among treatments at 20–40 cm depth, exhibiting a pattern of MCP > PMC > CCC > CPM, and with MCP showing a significant increase of 18.41% and 7.21%, respectively, compared to the CCC treatment. The PMC treatment showed the lowest fungal abundance and diversity, with a decline of 21.15% and 13.56, respectively. Summarily, in all treatments, the abundance and diversity of bacteria decreased with increasing soil depth, while the diversity of fungal communities grew with no significant change in abundance. Crop rotation enhanced the abundance of the bacterial community but diminished its diversity. In the 0–20 cm soil layer, the alpha diversity of the microbial community of legume crops (peanut) was lower than that of graminaceous crops (maize, millet).

#### 3.4.2. Composition of the Bacterial and Fungal Communities

**Figure 5:**
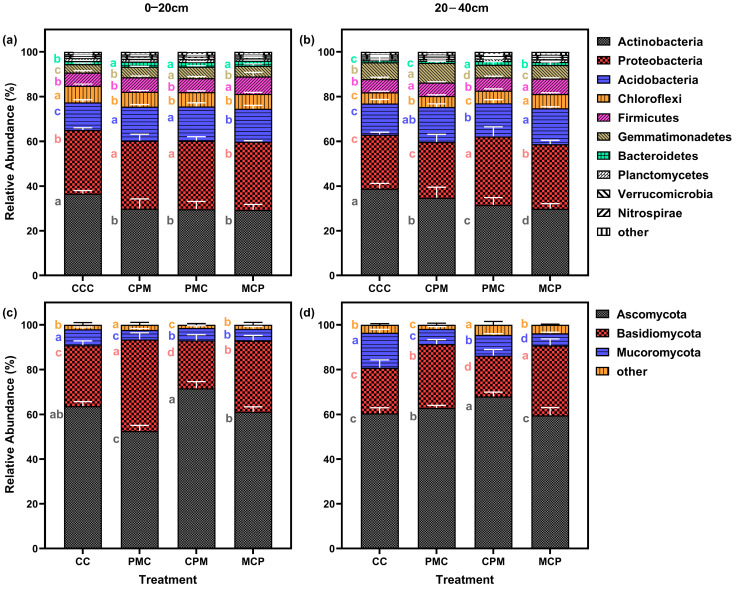
Effects of different cropping systems on the composition of the bacterial (**a**,**b**) and fungal (**c**,**d**) communities. A total of 0–20 cm (**a**,**c**) and 20–40 cm (**b**,**d**) soil depth. CCC is for 2020–2022 continuous cropping treatment, CPM, PMC, and MCP are for 2020–2022 rotation treatment, where C is for maize, P is peanut, and M is for millet. The bars represent the mean ± SE, n = 15 replicates. Lowercase letters to the left of the error line (a–c) indicate differences between treatments for the same colony at the same soil depth (*p* < 0.05).

Soil samples from continuous and rotational cropping allowed the annotation of 37 bacterial phyla and 27 fungal phyla. After selecting the top 20 phyla in terms of relative abundance and excluding those with less than 1% abundance, 10 bacterial (Figure 5a,b) and 3 fungal (Figure 5c,d) phyla remained. Actinobacteriota, Proteobacteria, Acidobacteriota, Chloroflexi, and Firmicutes, were the respective first five dominant phyla of bacteria at depths of 0–20 cm as well as 20–40 cm. The last dominant phylum showed a change from the 0–20 cm soil layer for Bacteroidetes and Gemmatimonadetes in the 20–40 cm soil layer, with the relative abundance of dominant phyla accounting for 93.43–94.57% and 94.21–95.41% of the total. Compared to the CCC treatment, the abundance of Actinobacteriota in the rotation treatment decreased by an average of 19.13% and 17.55% in the two soil layers.

Proteobacteria and Acidobacteriota increased by an average of 7.36–16.74% and 11.27%–22.23% in the two soil layers, whereas Chloroflexi was not significantly different. When the rotation treatments were compared, there was no significant difference among the three treatments for each dominant phylum in the 0–20 cm soil layer. However, the CPM treatment showed a rise of 10.08–16.37% and 44.04–55.43% for Actinobacteriota and Gemmatimonadetes in the 20–40 cm layer, while the Proteobacteria reduced by 15.50–22.63%. With increasing soil depth, the abundance of Actinobacteriota and Acidobacteriota grew by an average of 7.53% and 4.92%, respectively, while the abundance of Proteobacteria, Chloroflexi, and Firmicutes decreased by an average of 9.37%, 19.16%, and 6.51%, respectively.

At 0–20 cm and 20–40 cm depth, the dominant phyla for fungi were Ascomycota, Basidiomycota, and Mortierellomycota, whose relative abundance accounted for 95.52–98.89% of the total. Compared to the CCC treatment, the relative abundance of Ascomycota in the 0–20 cm soil layer grew by 14.23% in the CPM treatment but declined by 15.31% in the PMC treatment. However, the abundance of Basidiomycota increased by 48.43%. The Mortierellomycota abundance was lower in all treatments than in the CCC treatment (10.45–47.39%). Ascomycota and Basidiomycota abundance was higher than in CC treatments by 2.16–58.13%, while Mortierellomycota was lower than in CCC treatments by 37.63–66.56% in the three rotation treatments in the 20–40 cm soil layer.

Comparing the rotation treatments among each other showed that Ascomycota and Mortierellomycota were higher in CPM treatments than in PMC and MCP treatments. Basidiomycota was lower in the 0–20 cm and 20–40 cm CPM treatments than in PMC and MCP treatments. The percent abundance of Basidiomycota declined with increasing soil depth, while Ascomycota and Mortierellomycota did not significantly differ.

#### 3.4.3. PCA Analysis of Bacterial and Fungal Communities

**Figure 6:**
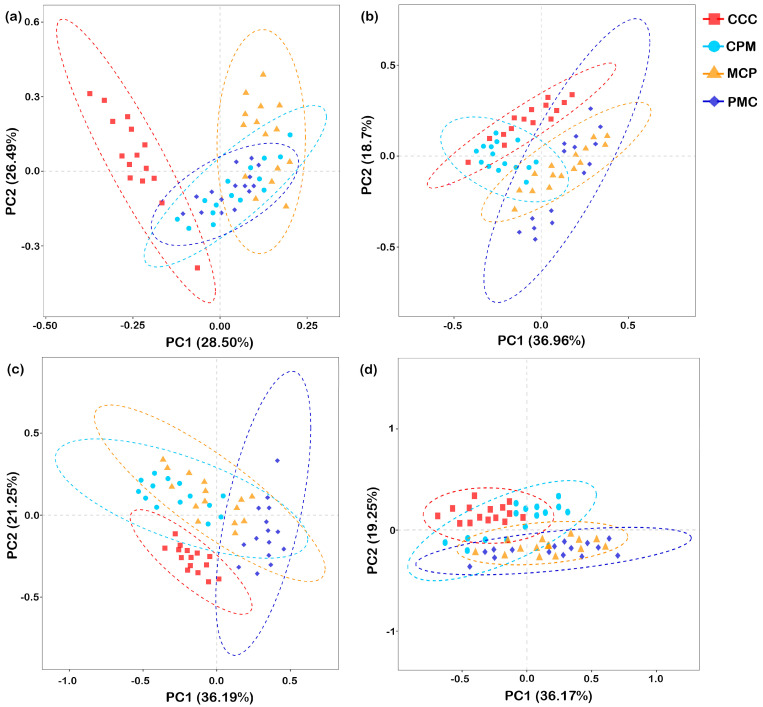
Principal component analysis of the soil microbial communities. Bacteria, 0–20 cm (**a**) and 20–40 cm (**b**) soil depth, fungal, 0–20 cm (**c**), and 20–40 cm (**d**) soil depth. CCC is for 2020–2022 continuous cropping treatment, CPM, PMC, and MCP are for 2020–2022 rotation treatment, where C is for maize, P is peanut, and M is for millet. n = 15 replicates. The dashed ellipse represents the 95% confidence interval.

Beta diversity analysis was performed based on Aitchison distance. PCA was chosen to compare the degree of similarity that existed in terms of species community diversity among the different samples (Figure 6a–d). For each treatment at depths of 0–20 cm and 20–40 cm, the first principal component (PC1) explained 28.50% and 36.96%, while the second principal component (PC2) explained 26.49% and 18.70% similarity at the bacterial gate level (97% similarity), respectively. The rotational crop treatments in the 0–20 cm soil layer were segregated from the CC treatments in both PC1 and PC2. The CPM and PMC bacterial communities were similar and concentrated in quadrants three and four, but the MCP treatment segregated from it in PC2 and concentrated in the second quadrant. The CCC treatments in the 20–40 cm soil layer had bacterial communities similar to the CPM treatments but segregated from the MCP and PMC in both PC1 and PC2.

At 0–20 cm and 20–40 cm depths, PC1 explained 36.19% and 36.17%, while PC2 explained 21.25% and 19.25% similarity at the fungal phylum level for each treatment, respectively. Separation of each of the rotation treatments from the CCC treatments on PC2 occurred in both 0–20 cm and 20–40 cm soil layers. In the 0–20 cm soil layer, the PMC was segregated from the CCC, CPM, and MCP treatments on PC2. In the 20–40 cm soil layer, the fungal communities were similar across treatments in the rotation and concentrated in the third and fourth quadrants. In conclusion, as compared to the CCC treatment, crop rotation changed the structure of the soil bacterial and fungal communities. The soil bacterial community structure differed significantly (*p* < 0.05) between crop rotation treatments with crop change. The fungal community of legume crops varied significantly from that of graminaceous crops from 0 to 20 cm, but the difference was not significant from 20 to 40 cm.

#### 3.4.4. Co-Occurrence Networks and Ecological Clustering of Bacteria and Fungi

**Figure 7:**
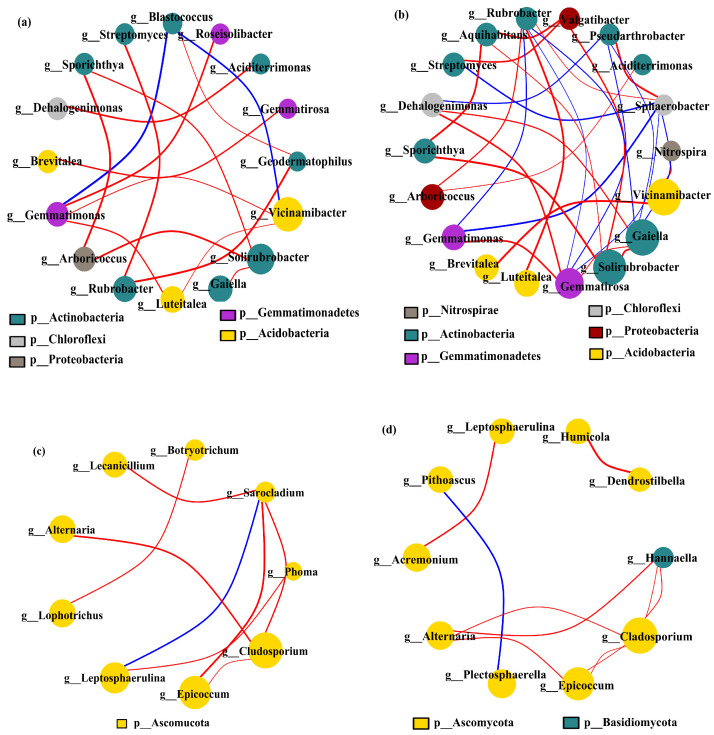
Co-occurrence network model of bacterial (**a**,**b**) and fungal (**c**,**d**) communities under varied cropping patterns. Top layer 0–20 cm (**a**,**c**) (matching CCC, CPM, PMC, and MCP by 97% similarity) and deeper 20–40 cm (**b**,**d**) (matching CCC, CPM, PMC, and MCP by 97% similarity) soil depth. The circles indicate different species (genus level), with size denoting the average abundance. The blue and red lines denote the positive and negative correlation, respectively, with thickness associated with the degree of correlation.

Under different cropping scenarios, genus-level covariate network models were constructed of the dominant soil microbial communities in the 0–20 cm and 20–40 cm soil layers (matching CCC, CPM, PMC, and MCP by 97% similarity), excluding unnamed bacterial genera. Vicinamibacter, Gaiella, Solirubrobacter, and Gemmatirosa were dominant bacteria, while Gladosporium and Epicoccum were predominant fungi (Figure 7a–d). The most prevalent bacterial genera were Actinobacteria, while the dominant fungal genera were mainly Ascomycota. Comparing the bacterial and fungal networks between the two soil layers, it was found that the number of soil microbial nodes and edges rose in the 20–40 cm soil layer as compared to the 0–20 cm soil layer. This suggests that the relationships between genera tend to be complicated due to the increased diversity of soil microbial communities in the 20–40 cm soil layer. The negative correlation was more predominant than the positive one for both soil layers. The proportion of negative correlation was 88.24% and 43.33% for bacteria and 87.50% and 90.00% for fungi, respectively.

In conclusion, the species diversity of the soil microbial community increased with the depth of the soil layer. The competitive relationship between species was weakened, while the synergistic ability was strengthened.

#### 3.4.5. Correlation between Chemical Properties and Microbial Communities

**Figure 8:**
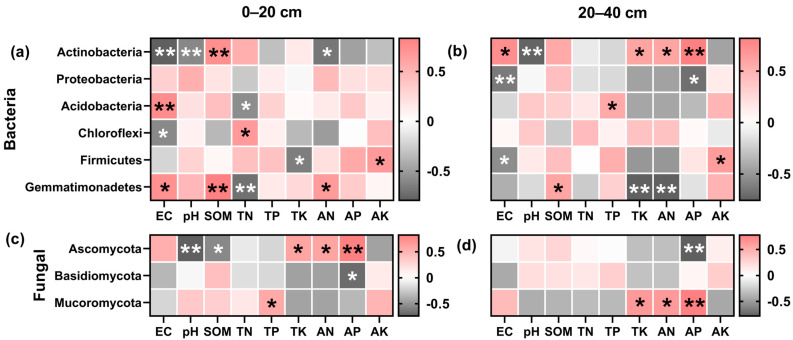
Correlation analysis between soil dominant bacteria and environmental factors. Bacteria (**a**,**b**) and fungal (**c**,**d**), 0–20 cm (**a**,**c**), and 20–40 cm (**b**,**d**) soil depth, with red segments being positively correlated and dark grey segments being negatively correlated, and asterisks indicating a significant correlation (* *p* < 0.05, ** *p* < 0.01). Soil organic matter (SOM), total nitrogen (TN), total phosphorus (TP), total potassium (TK), available nitrogen (AN), rapidly available phosphorus (AP), and available potassium (AK).

The abundance of predominant bacteria can be greatly influenced by variations in the soil environment. As seen in Figure 8a,b, the dominant bacteria in the different plough depths had varying interrelationships with environmental factors. Actinobacteria exhibited a negative correlation (*p* < 0.01) with pH and EC in both the plough layers, but a significant positive correlation with EC in the deep soil layer (20–40 cm). Actinobacteria were positively correlated with SOM on the top but with AP in the deeper layers. Proteobacteria exhibited a negative association with EC and AP in the deep soil layer.

Gemmatimonadetes and Acidobacteria demonstrated a positive association with soil EC and SOM in the topsoil layer. The rise in the Gemmatimonadetes abundance in the deep soil layer led to reduced TK and AN content. Similarly, the upsurge in Chloroflexi and Firmicutes abundance led to a deterioration of the soil environment and a drop in nutrient content. In the topsoil layer, a positive correlation of Ascomycota with TK, AN, and AP was observed, contrary to a negative association with SOM and pH (Figure 8c). The AP content of the soil decreased as Ascomycota abundance rose in the deep soil layer (Figure 8d). The Mortierellomycota ampleness raised soil nutrients in the deep soil. Basidiomycota showed a significant negative correlation (*p* < 0.05) with AP in the topsoil layer.

#### 3.4.6. Functional Analyses of Soil Microbial Communities

For each treatment, the gene functions of the microorganisms were analyzed according to the information system of the Kyoto Encyclopedia of Genes and Genomes (KEGG) database. The KEGG orthology (KO), pathways, and Enzyme Commission (EC) insights were obtained. The abundance of each functional category was calculated based on operational taxonomic unit (OTU) abundance. Three gene functions related to nitrogen metabolism, phosphorus metabolism, and plant pathogens were screened. As shown in Table 2, the three-year rotation treatments increased N metabolism and P metabolism functions in the 0–20 cm and 20–40 cm soil layers by 8.61–65.77% and 44.73–88.65%, respectively, compared to the CCC treatment. Plant pathogens decreased by 18.48–27.81% (*p* < 0.05). Among the crop rotation treatments, soil N metabolism and P metabolism functions in the 0–20 cm soil layer were significantly higher in the PMC (peanut) treatment than in the CPM and MCP treatments, with an increase of 14.18–29.64%. However, there was no significant difference between CPM and MCP.

In the 20–40 cm soil layer, the soil N metabolism function exhibited a pattern of PMC > MCP > CPM, while it was CPM > MCP > PMC for the P metabolism function. There was no significant difference (*p* < 0.05) in the abundance of plant pathogens in the two soil layers for each crop rotation treatment. Overall, crop rotation enhanced soil nutrient metabolism capacity and reduced pathogen abundance, and legume crops had a greater nutrient metabolism capacity for surface soils than grasses crops.

### 3.5. Crop Yield and Economic Benefits

Table 3 shows that rotational settings boosted the 2020–2022 average maize yield (CPM) by 32.07% as compared to continuous cropping maize (CCC). Similarly, in comparison to CCC treatment, CPM, PMC, and MCP treatment raised income by an average of 22.25%, income increased by USD 346.82 per hectare, and output/input increased by 10.26%.

### 3.6. Structural Equation Modelling

**Figure 9:**
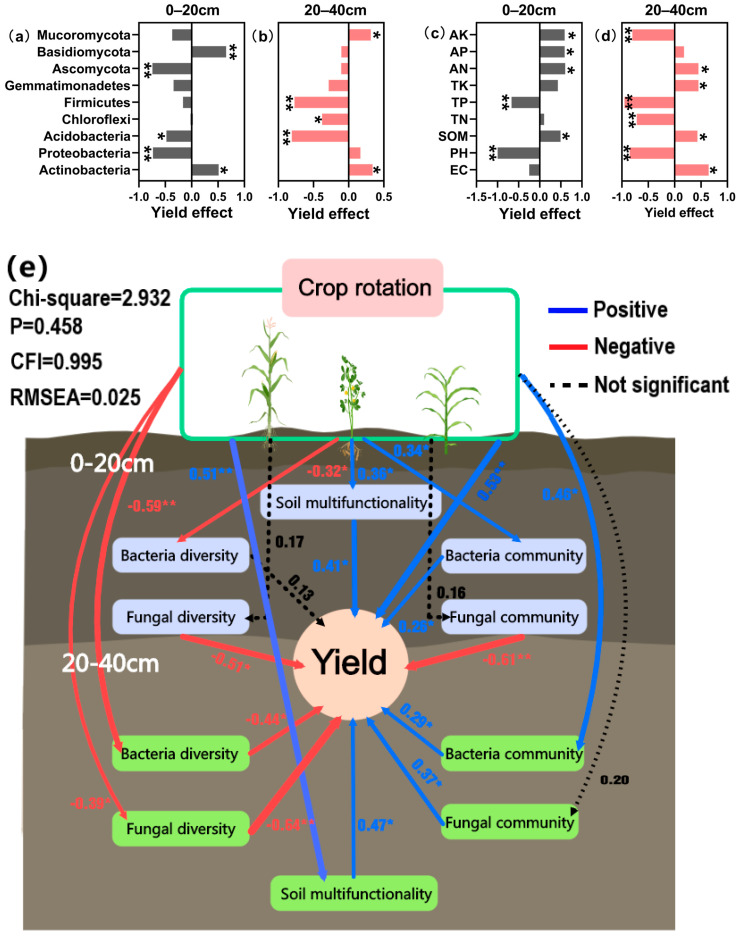
The effect of dominant microorganisms (**a**,**b**) and soil chemistry properties (**c**,**d**) on yield. Soil organic matter (SOM), total nitrogen (TN), total phosphorus (TP), total potassium (TK), available nitrogen (AN), rapidly available phosphorus (AP), and available potassium (AK). Structural equation model (**e**), positive associations are indicated by blue arrows, while negative relationships are depicted by red arrows, and black dashed arrows indicate no significant correlation between the two. Statistical significance is denoted by asterisks (* *p* < 0.05; ** *p* < 0.01). CFI denotes the comparative fit index and RMSEA denotes the root mean square error of approximation.

The findings of structural equation modelling (Figure 9a) reveal that the rise in soil bacterial and fungal abundance in different tillage levels (0–20 cm and 20–40 cm) could significantly enhance yield, but the increase in their diversity reduced crop yield. Crop rotation significantly increased the abundance of the soil microbial community but decreased its diversity in the range of 7.74–21.15% (*p* < 0.05). Additionally, crop rotation raised the community function of soil bacteria and fungi, which in turn improved the yield.

Figure 9b,c shows the influence of dominant bacteria on yield in the different plough layers. The increase in the relative abundance of Actinobacteria in both soil layers enhanced yield, whereas the enrichment in the relative abundance of Acidobacteria in both layers declined the yield. Furthermore, enrichment of Firmicutes and Chloroflexi among bacteria in the 20–40 cm soil layer and Ascomycota among fungi in the 0–20 cm soil layer had varied degrees of detrimental effects on yield (*p* < 0.05).

Figure 9d,e illustrates the effect of soil chemical properties on yield in different tillage layers. The findings reveal that the increase of SOM and soil-available nutrient content could boost crop yield, but the enrichment of available potassium content in the 20–40 cm soil layer declined the yield. The soil pH and TP also impacted the yield adversely. The relationship between soil TN content and yield varied in different ploughing layers, as evidenced by an increase in yield with 0–20 cm enrichment, but a reduction in the case of 20–40 cm enrichment.

## 4. Discussion

### 4.1. Significantly Differential Impact of Varying Treatments on Soil Chemical Attributes

A substantial increase in soil pH and salt ion concentration leads to the deterioration of crop survival environment and soil nutrient deficiency, which largely hinders crop growth and development [29]. The results of this study show that crop rotation can mitigate its negative effects on crops. The PCA of soil factors revealed that the main factors affecting wind–sand saline soils were EC, pH, AK, SOM, and AN, and crop rotation had a considerable impact on all five of these factors [30]. The findings show that after three years of crop rotation, EC and SOM content increased, whereas pH, AK, AN, and AP content decreased in the 0–20 cm and 20–40 cm saline soil layers. Crop rotation between legumes and grasses can maximize soil nutrient utilization in different plough layers and reduce nutrient losses. The inherent nitrogen capacity of legumes increases EC and nutrients in the soil, improves soil buffering capacity, and reduces the pH of sandy saline–alkaline soils [31].

Crop rotation can balance soil nutrients, effectively reduce the inhibitory effect of soil mono elements, improve soil metabolism, and subsequently increase SOM content. The acidifying functional groups released during the oxidation of organic matter to SOM may also be the reason for the decrease in pH in sandy saline–alkaline soils [32]. In addition, SOM has a strong adsorption capacity and contributes about 20–70% to soil EC [33]. It has a buffering effect, retards the movement of deep salt ions to the surface soil, neutralizes soil alkalinity, improves soil nutrition, and promotes an increase in EC.

Crop rotation can effectively boost crop nutrient uptake and increase soil fertility and nutrient use efficiency [34], which is consistent with the results of this study. In the present work, the contents of TN, TP, and TK in the 0–20 cm soil layer increased, but the contents of AK, AN, and AP decreased, while soil fertility and nutrient use efficiency improved. Moreover, the initial stage of SOM formation activates the oxidation of volatiles and surface functional groups and blunts the interaction of salt ions with the soil to establish a protective mechanism that enhances the ability of the soil to absorb nutrients [35,36].

### 4.2. Higher Abundance of Microbial Communities in Rotational Treatment

Recently, there was an exponential surge in research into the effects of agrotechnical methods on soil microbial communities [37]. However, determining exactly which factors influence the structure and diversity of microbial communities in saline soils remains difficult. This study found that the abundance of microbial communities in soils of different crop rotation tillage treatments was higher than that of continuous cropping. This is in agreement with the findings of Xu et al. (2024) [38]. The relative abundance of bacterial phylum levels was higher in rotated soils than in continuous cropping, while for fungi, it was rotated less than in continuous cropping. This indicated that the composition of bacterial communities in rotated soils was significantly affected by changes in tillage practices. The findings of PCA showed that the microbial communities of the two tillage systems differed significantly, which may be because the bacterial composition was affected by crop rotation [39].

Actinobacteria and Mortierellomycota can effectively increase soil nutrient content after crop rotation. This can be attributed to the mutual promotion of soil urease and phosphate reductase activities, which boosts nitrogen and phosphate metabolism, but is negatively correlated with plant pathogen abundance, reducing the number of soil pathogens [40,41]. Furthermore, soil permeability is improved after the rotation of legumes and grasses, as both Actinobacteria and Mortierellomycota are aerobic bacteria and can fix atmospheric nitrogen under low oxygen pressure [42]. By constructing the cooccurrence network model, it was found that the number of soil bacterial edges was greater in 20–40 cm tillage compared to 0–20 cm. The interrelationships between the phyla tend to be complicated; with the increase in species richness, the competitive relationship between the species is weakened. Thus, crop rotation improved the survival environment of the dominant bacterial phyla in the soil, which in turn led to the enhancement of their symbiotic relationship and the weakening of their competitive relationship [43].

### 4.3. Higher Grain Yields in Rotation Treatment

The effects of saline soils on crops are primarily due to the high salt concentration, pH, and ionic toxicity in the soil. This leads to excessive uptake of ions by the plant and increased osmotic pressure, disrupting the plant’s water homeostasis, upsetting its normal nutrient balance, and inhibiting growth [44]. In this study, crop rotation could effectively reduce soil pH and increase soil SOM content. The soil pH was negatively correlated with yield, while SOM and soil-available nutrients could be positively correlated with yield. This is due to enhanced nutrient availability, greater competition with toxic ions in the soil, reduced Na+ uptake by the crop, and subsequent low toxicity of saline ions, which promotes crop growth and development [45]. In addition to being rich in carbon, SOM can be used as a direct source of nutrients or contribute to plant growth and yield through cation exchange, surface interactions, and water utilization processes [46].

Structural equation modelling analysis showed that crop rotation increased microbial community richness but decreased its diversity. The increase in richness increased microbial community functioning, which in turn improved crop yield. Enrichment of Actinobacteria in bacteria and Basidiomycota and Mortierellomycota in fungi increased crop yield. All three of these dominant groups can be identified as a dissimilatory nitrate reduction to ammonium (DNRA) community [47]. Crop rotation can reduce ammonium levels, lowering N losses through ammonia volatilization and promoting AN accumulation. Basidiomycota can produce chitinase, and the products of organic N degradation by chitinase can be considered a source of available N [48]. Furthermore, Basidiomycota was reported to suppress plant pathogens, which may contribute to plant growth and nutrient accumulation through increased root secretions [49]. Ascomycota enrichment can lead to crop yield reduction. Ascomycota is a large and complex group of fungi that includes many phytopathogenic bacteria such as Chaetomium and Fusarium, which can cause root rot in crops. Thus, its increased abundance may predict a possible increase in the incidence of crop diseases [50].

## 5. Conclusions

In summary, the obtained findings confirmed the hypothesis speculated during the trial. Crop rotation can reduce the pH (−2.36%) of saline soil and increase SOM (13.41–34.12%) and nutrient content. Therefore, crop rotation cultivation patterns can be an effective measure to improve saline soil and enhance soil fertility. The correlation analysis of crop yield with soil-dominant microbes and environmental factors identified the mechanistic ways of crop rotation to increase yield. Crop rotation increases the abundance and decreases the diversity of soil microbial communities. This alters the way their bacterial and fungal communities are composed, which in turn enhances bacterial and fungal community function. The insights generated from this study will have pertinent implications, especially for saline lands in establishing diversified crop rotation as a viable approach to raise crop yields and economic benefits by improving the function of microbial communities and balancing soil nutrients.

## Figures and Tables

**Table 1:** Principal component analysis of soil factors.

Ingredient	Initial Eigenvalue			Extracting the Sum of Squared Loads			Score
	Total	Percentage of Variance	Accumulation %	Total	Percentage of Variance	Accumulation %	
EC	3.19	35.46	35.46	3.19	35.46	35.46	0.71
pH	2.58	28.65	64.11	2.58	28.65	64.11	0.23
AK	1.47	16.51	80.63	1.49	16.51	80.63	0.28
SOM *	0.85	9.48	90.10				0.07
AN	0.52	5.98	96.08				−0.09
AP	0.12	1.38	97.46				−0.46
TN	0.11	1.21	98.67				0.89
TK	0.07	0.76	99.43				−0.93
TP	0.05	0.57	100.00				0.83

* Soil organic matter (SOM), total nitrogen (TN), total phosphorus (TP), total potassium (TK), available nitrogen (AN), rapidly available phosphorus (AP), and available potassium (AK).

**Table 2:** Statistics of the functional abundance of soil genes from 0 to 20 cm and 20 to 40 cm.

Soil Layer (cm)	Treatment	N Metabolism	P Metabolism	Plant Pathogen
0–20	CCC *	0.46 ± 0.00 ^c^	0.10 ± 0.00 ^b^	0.15 ± 0.00 ^a^
	PMC	0.65 ± 0.04 ^a^	0.17 ± 0.04 ^a^	0.12 ± 0.02 ^b^
	CPM	0.50 ± 0.02 ^b^	0.15 ± 0.05 ^ab^	0.11 ± 0.03 ^b^
	MCP	0.50 ± 0.02 ^b^	0.14 ± 0.04 ^ab^	0.12 ± 0.01 ^b^
20–40	CCC	0.32 ± 0.01 ^c^	0.08 ± 0.01 ^c^	0.16 ± 0.00 ^a^
	PMC	0.53 ± 0.03 ^a^	0.13 ± 0.02 ^b^	0.13 ± 0.01 ^b^
	CPM	0.42 ± 0.12 ^b^	0.16 ± 0.01 ^a^	0.13 ± 0.01 ^b^
	MCP	0.44 ± 0.00 ^b^	0.14 ± 0.01 ^ab^	0.12 ± 0.01 ^b^

* CCC is for 2020–2022 continuous cropping treatment, CPM, PMC, and MCP are for 2020–2022 rotation treatment, where C is for maize, P is peanut, and M is for millet. n = 15 replicates. Superscript lowercase letters (a–c) indicate significant differences between the different treatments (*p* < 0.05).

**Table 3:** Crop yield and economic benefits.

Year	Patterns	Crop Types	Input (USD ha^−1^)	Yield (t ha^−1^)	Price (USD kg^−1^)	Output (USD ha^−1^)	Net Output (USD ha^−1^)	Output/Input (%)
2020	CPM	Maize	1733.68	12.94	319.93	4141.14	2407.46	238.86
	PMC	peanut	1902.33	2.65	1130.78	2994.08	1091.75	157.39
	MCP	millet	1559.65	3.89	661.92	2573.08	1013.43	164.98
	CCC *	Maize	1733.68	9.85	319.93	3151.29	1417.61	181.77
2021	CPM	Maize	1842.34	13.66	340.61	4652.75	2810.40	252.55
	PMC	peanut	2111.25	3.03	1185.94	3591.47	1480.22	170.11
	MCP	millet	1657.56	4.73	689.50	3259.96	1602.40	196.67
	CCC	Maize	1842.34	9.59	340.61	3267.13	1424.78	177.34
2022	CPM	Maize	2096.08	13.01	372.33	4845.12	2749.04	231.15
	PMC	peanut	2275.35	3.44	1379.00	4740.31	2464.96	208.33
	MCP	millet	1820.28	5.76	581.94	3352.49	1532.21	184.17
	CCC	Maize	2096.08	10.56	372.33	3930.29	1834.21	187.51
Average	CPM	-	1890.75	13.21	344.75	4546.29	2655.68	240.45
	PMC	-	2096.36	3.04	1231.45	3775.29	1678.93	180.09
	MCP	-	1679.21	4.79	643.99	3061.79	1382.72	182.34
	CCC	Maize	1890.75	10.00	344.75	3449.57	1558.96	182.44

* CCC is for 2020–2022 continuous cropping treatment, CPM, PMC and MCP are for 2020–2022 rotation treatment, where C is for maize, P is peanut and M is for millet. n = 3 replicates. Currency conversion: JPY 1 ≈ USD 0.1379 (29 May 2024).

## Data Availability

The datasets generated and analyzed during the current study are available from the corresponding author upon reasonable request.

## Abbreviations

**Abbreviations**	**Explanations**
CK	2019 soil samples
CPM	Maize–peanut–millet rotations
PMC	Peanut–millet–maize rotations
MCP	Millet–maize–peanut rotations
CCC	Maize continuous cropping
SOM	Soil organic matter
TN	soil total nitrogen
TP	total phosphorus
TK	total potassium
AN	available nitrogen
AP	rapidly available phosphorus
AK	available potassium
